# Light-to-Heat Conversion of Optically Trapped Hot Brownian Particles

**DOI:** 10.1021/acsnano.3c07086

**Published:** 2023-12-04

**Authors:** Elisa Ortiz-Rivero, Sergio Orozco-Barrera, Hirak Chatterjee, Carlos D. González-Gómez, Carlos Caro, María-Luisa García-Martín, Patricia Haro González, Raúl A. Rica, Francisco Gámez

**Affiliations:** † Nanomaterials for Bioimaging Group, Departamento de Física de Materiales, & Instituto de materiales Nicolás Cabrera & Institute for Advanced Research in Chemical Sciences,, Universidad Autónoma de Madrid, Madrid 28049, Spain; ‡ Universidad de Granada, Nanoparticles Trapping Laboratory, Research Unit Modeling Nature (MNat) and Department of Applied Physics, 18071 Granada, Spain; § Universidad de Málaga, Department of Applied Physics II, 29071 Málaga, Spain; ∥ Biomedical Magnetic Resonance Laboratory-BMRL, Andalusian Public Foundation Progress and Health-FPS, 41092 Sevilla, Spain; ⊗ Biomedical Research Institute of Málaga and Nanomedicine Platform (IBIMA-BIONAND Platform), University of Málaga, C/Severo Ochoa 35, 29590 Málaga, Spain; ∇ Biomedical Research Networking Center in Bioengineering, Biomaterials & Nanomedicine (CIBER-BBN), 28029 Madrid, Spain; ○ Department of Physical Chemistry, Universidad Complutense de Madrid, 28040 Madrid, Spain

**Keywords:** optical tweezers, hybrid nanostructures, heat generation, nanothermometry, hot Brownian motion

## Abstract

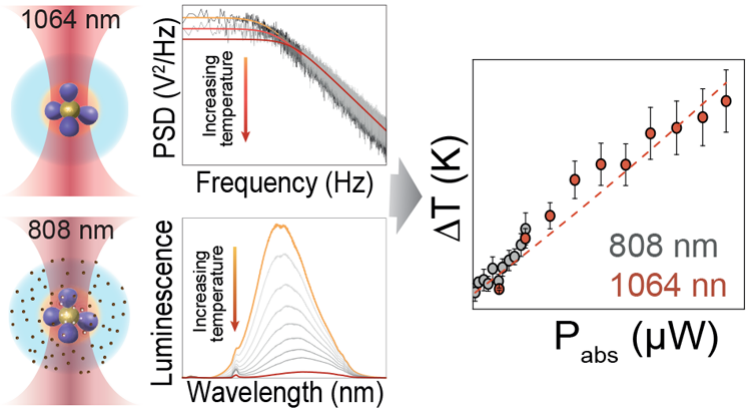

Anisotropic hybrid nanostructures stand out as promising therapeutic agents in photothermal conversion-based treatments. Accordingly, understanding local heat generation mediated by light-to-heat conversion of absorbing multicomponent nanoparticles at the single-particle level has forthwith become a subject of broad and current interest. Nonetheless, evaluating reliable temperature profiles around a single trapped nanoparticle is challenging from all of the experimental, computational, and fundamental viewpoints. Committed to filling this gap, the heat generation of an anisotropic hybrid nanostructure is explored by means of two different experimental approaches from which the local temperature is measured in a direct or indirect way, all in the context of hot Brownian motion theory. The results were compared with analytical results supported by the numerical computation of the wavelength-dependent absorption efficiencies in the discrete dipole approximation for scattering calculations, which has been extended to inhomogeneous nanostructures. Overall, we provide a consistent and comprehensive view of the heat generation in optical traps of highly absorbing particles from the viewpoint of the hot Brownian motion theory.

## 1 Introduction

Multicomponent nanoparticles (MCNPs) can be defined as single structures that combine the properties of at least two different materials at the nanoscale.^1−4^ A myriad of applications of à la carte MCNPs have occupied the limelight of different research fields throughout the last years because of the virtually endless material combinations that confer specific features designed for each potential purpose.^5^ For instance, their relevance in biomedical applications has taken off due to their ability to act both as a dual agent for multimodal contrast and/or for combined/synergic therapies.^6,7^ In this context, materials engineering has developed specific routes for costuming a wide plethora of on-demand nanocomposites whose shape, functionalization, and spectroscopic attributes of the surface plasmon resonance (SPR) provide distinctive physical and biocompatibility features that are key for real-world applications in nanomedicine.^8,9^ The so-called inductive synthesis has emerged in this field as an intriguing branch of nanoscience that fosters the adaptation of the morphology of nanoparticles to achieve specific biological responses.^10^ In particular, MCNPs comprised of a light-absorbing material (i.e., gold) and magnetic moieties can be conceived as agents for hyperthermia treatments by dint of their efficient heat-conversion effect under both alternating magnetic fields and near-infrared light.^11−14^ It then becomes apparent that measuring temperatures at the nanoscale and understanding the photothermal conversion of light-absorbing hybrid nanoparticles at the single-particle level have become central issues in applied physics and materials engineering.

A captivating approach to manipulate, control, and measure properties of isolated nanostructures is embodied by the continuously upgraded technology of optical tweezers.^15^ Even if challenging, trapping absorbing nanoparticles, in particular those made of noble metals that feature plasmonic resonances, has been recently optimized.^16−19^ Particularly, optical tweezers constitute an alternative route toward the microscopic comprehension of heat generation by isolated nanoparticles by monitoring changes in the properties of the heating source^20,21^ or its surroundings.^22^ Some previous works were devoted to evaluating the local temperature in optically trapped nanoparticles using viscosity variations of the surrounding media upon laser heating^23−25^ or by the indirect monitorization of the temperature-dependent emission of a nanoprobe.^26^ In the last case, the eventual influence of the neighboring nanothermometers on the absorption, the center of mass motions, and the light-to-heat conversion efficiency of the trapped particle is a question that should be considered. But a word of caution: temperature is a many-particle property, and therefore it is a blurry-defined concept from statistical mechanics grounds in single-particle systems far from equilibrium, jeopardizing the validity of traditional Brownian motion prescriptions. In consequence, another point of interest is whether the measured “temperature” resembles the classic interpretation of surface or internal temperatures or if it is just a value that averages local thermal fluctuations of the solvent bath within the trap volume enabling the description of the particle dynamics within the so-called hot Brownian motion (HBM) theory.^27−30^ To address these points, experimental and theoretical results on the heat generation and local temperature of a light-absorbing multicomponent nanoparticle under optical trapping conditions are accomplished here.

## 2 Results and Discussion

### 2.1 Characterization of the MCNPs

#### 2.1.1 Physicochemical Characterization

**Figure 1:**
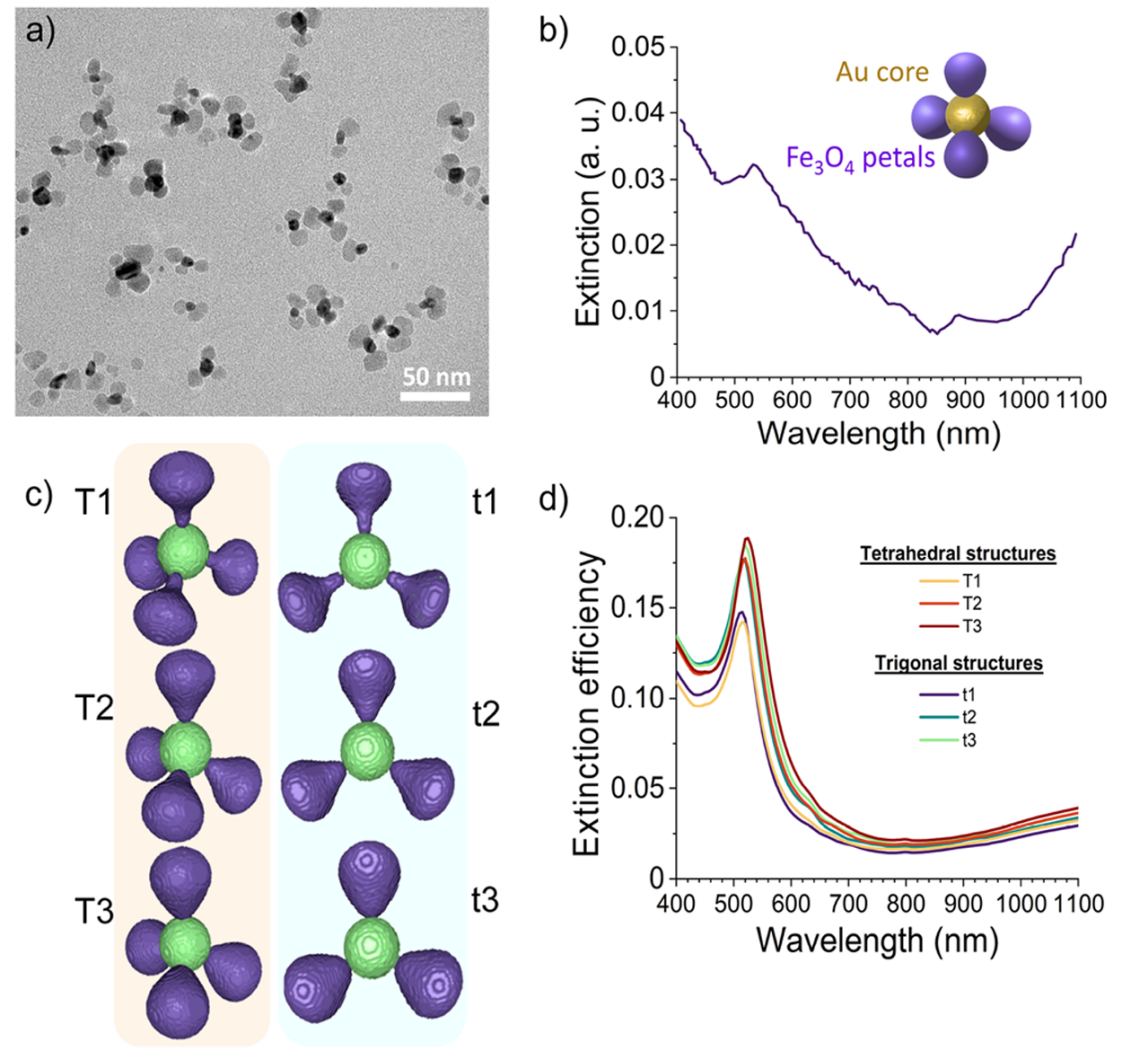
Physicochemical characterization of the hybrid nanoparticles. (a) TEM image of the synthesized nanoflowers. (b) Extinction spectrum of a diluted suspension of the nanoflower suspensions. The inset represents a schematic model of the composition of the nanoflowers. (c) Theoretical models for the calculation of the scattering profiles of the nanoflowers: models of the whole nanoflowers with 4 (left column) and 3 (right column) petals, for β = 1.5 (T1, t1), β = 2.5 (T2, t2), and β = 5 (T3, t3). (d) Extinction efficiencies for all the structures calculated with DDA.

In this work, we faced thermometric measurements on hybrid nanoflowers (NFs) synthesized by the multinucleation of magnetite globular petals on spherical gold seeds, as reported in reference^31^ and detailed in the Experimental Section. Transmission electron microscopy (TEM) was employed to characterize the MCNP morphology and the size distribution of the moieties of each component. The characteristic shapes are presented in the micrography of Figure 1a. The diameter of the different inorganic counterparts was ∼12 nm for both the Au core and Fe_3_O_4_ petals, respectively, as shown in the size histograms shown in Figure S1. Overall, and assuming an *effective* spherical shape, the TEM diameter of the whole nanoflower was ∼33 nm. After a PEGylation procedure, the colloidal stability of the aqueous suspension was confirmed by both the constancy of the hydrodynamic diameter (∼58 nm) for 1 month and the negative value of the nanoparticle ζ-potential of −14 mV that, according to DLVO theory, was expected to lead to stable suspensions.^32^ The extinction spectrum of a diluted suspension, shown in Figure 1b, presented a broad band with a maximum extinction wavelength of ∼535 nm because of the SPR of the gold core perturbed by the interaction with the magnetite globules. The light extinction increases in the NIR from ∼800 nm on. This behavior has been reported before in both experiments and Mie calculations and is ascribed to a charge-transfer absorption band in the magnetic moiety.^33^ The behavior of the long-tail extinction of the particles in the NIR envelops both the first and second biological windows^34^ that, together with their watery stability, enable their fundamental optical exploration as hyperthermia agents.^35^ From a purely optical viewpoint, these nanostructures combine the penetration depth and absorption of magnetite with the reflecting, heating, and trapping advantages of gold.

#### 2.1.2 Evaluation of the Absorption Cross Section from DDA Simulations

The synthesized nanoflowers were simulated by using parametric computer-aided design (CAD) modeling. To define the nanoflower geometry, the petals have been modeled using a piriform curve, which is parametrized just by one single parameter β (see Figure S2 in the Supporting Information). Sweeping the value of β from 1.5 to 6 with 0.5 step size, we developed parametric curves of the profiles of the petals, which were revolved in three dimensions to obtain the shapes of the petals, as shown in Figure S2. The flower shape has been conceived by the assignment of the petals around the core in both planar triangular and tetrahedral arrays for three selected shapes of petals having β values of 1.5, 2.5, and 5. Since the rise in β leads to a concomitant increase in the height of the petals, a scaling factor has been introduced to keep the size ratio of the core and petal within the limits of the experimental size range as shown by the histogram in Figure S1. The diameters of the core gold and the magnetite caps are set to 10 and 11.5 nm, respectively. Corresponding models for the triangular and tetrahedral arrays are shown in Figure 1c. Models T1 and t1 are the tetrahedral and triangular nanoflowers for β = 1.5. Similarly, models T2 and t2 show the tetrahedral and triangular nanoflower for β = 2.5, and models T3 and t3 show the tetrahedral and triangular nanoflower for β = 5, respectively. The composition assignment of the models for the particles synthesized in the present work has been modeled using a home-built Python code.^36^ In view of the symmetric structure of the composite nanoflower, the design of the code involves the superposition of magnetite petals with a gold core seeded inside. The interface between the two materials has been set to the dipole distance between the dipolar lattice considered for the scattering simulation considering the discrete dipole approximation (DDA) for gold core and magnetite petals. The scattering simulation has been performed using DDSCAT code to calculate scattering, absorption, and extinction cross-section at different wavelengths with corresponding near-field calculations within ±2 nm from the gold core in both *x*- and *y*-axes.^37^ The result illustrates a strong electromagnetic field appearing at the interface between the gold core and magnetite petals, as shown in Figure S3 for model T3. The appearance of a strong electric field indicates strong confinement of hot electrons at the material boundary, leading to processes like hot electron transfer and plasmon–phonon coupling, among others, which could be a future prospect of study. Finally, a plot of DDA-calculated extinction efficiencies for the six models is shown in Figure 1d). The patterns show a sharp peak at around 500 nm and a shoulder tail at around 1100 nm, depicting the inclusion of both gold and magnetite in the nanoflower, in agreement with the experimental extinction spectra shown in Figure 1b.

In order to relate the simulation information with the experiments performed at 1064 and 808 nm discussed below, we evaluated the average absorption cross-section, ⟨σ_abs_⟩, from the simulated absorption efficiency values obtained for the different *i* structures, *q*_abs_^*i*^, as ⟨σ_abs_⟩ = ⟨*q*_abs_^*i*^⟩π*a*_eff_^2^, where *a*_eff_ is the effective radius of scatterers.^38^ This information is given in Table 1. This approach accounts for the sample size and shape heterogeneity in an *effective* way.

**Table 1:** Calculated Values of Absorption Cross-Section and Absorption Efficiencies of the Selected Particle Models at the Wavelengths of Interest

	λ = 1064 nm		λ = 808 nm	
model	*q*_abs_ × 10^2^	σ_abs_ × 10^6^ μm^2^	*q*_abs_ × 10^2^	σ_abs_ × 10^6^ μm^2^
T1	3.02	9.22	1.66	5.09
t1	2.74	8.93	1.48	4.82
T2	3.40	11.1	1.93	6.30
t2	3.14	11.5	1.79	6.58
T3	3.66	12.2	2.16	7.21
t3	3.38	13.5	1.95	7.73

### 2.2 Nanothermometry of Trapped MCNPs

#### 2.2.1 Corner Frequency-Based Nanothermometry

**Figure 2:**
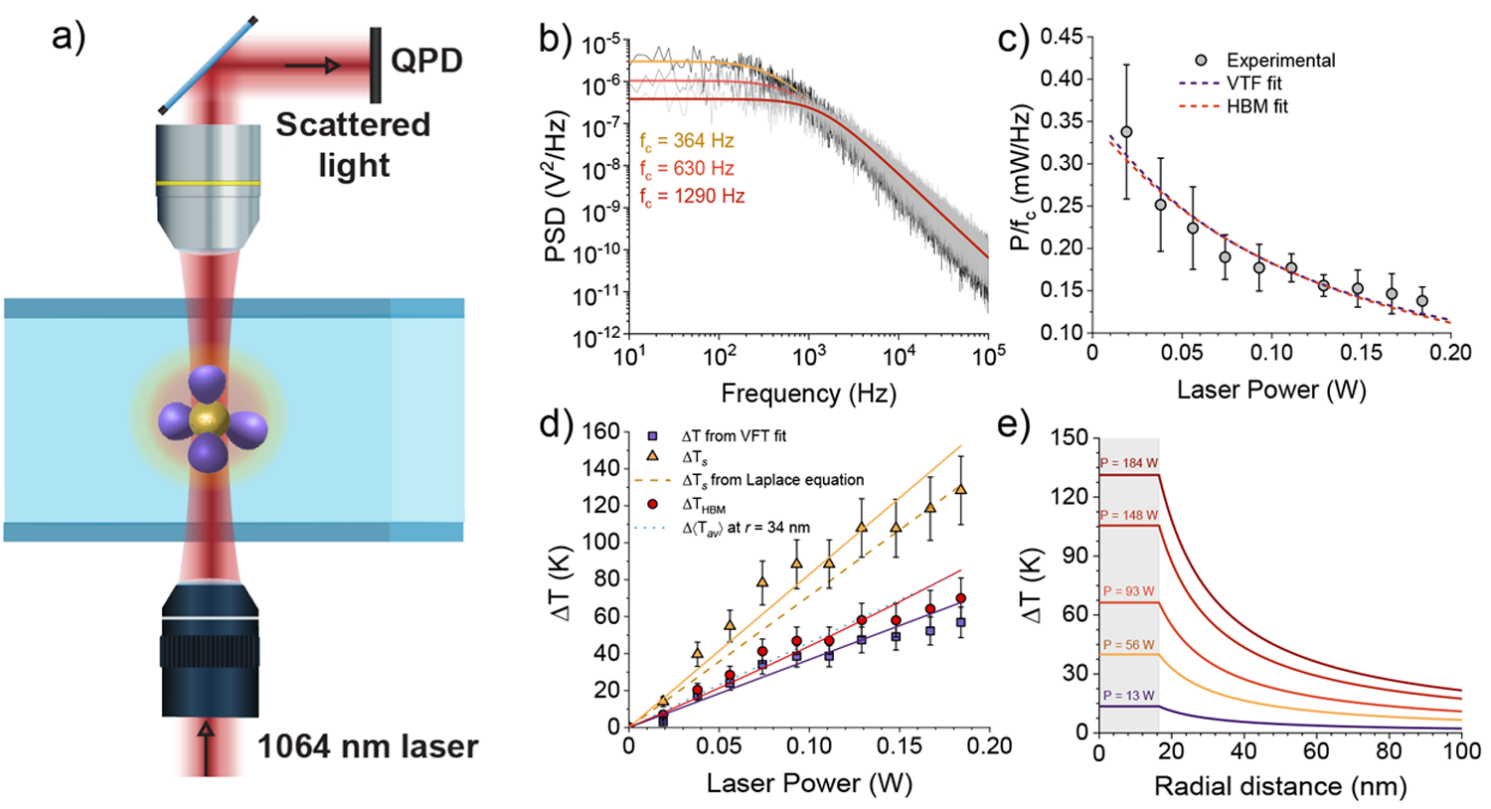
Corner frequency-based nanothermometry. (a) Schematic representation of the experimental setup. See Experimental Section for details. (b) Variation of the PSD with *P*. The black, dark gray, and light gray curves stand for the PSD of a trapped MCNP at laser powers of 74, 111, and 167 mW, respectively. The Lorentzian fits required to evaluate *f*_c_ are shown to exemplify the experimental procedure to estimate Δ*T* upon laser heating. See the main text for details. (c) Experimental values of the laser power-to-corner frequency *ratio* versus laser power. Error bars stand for the propagated error calculated from the standard deviation values evaluated over ten replicate measurements. The dashed lines denote the fit of the experimental data to eq 2 (violet line) and to the HBM theory (red line), respectively. (d) Temperature increments as a function of the laser power. Symbols stand for the experimental data obtained from eq 2 (violet squares), the surface temperature from HBM theory (yellow triangles), and the *effective* HBM theory (red circles), respectively. The continuous yellow and violet lines denote the results from the fittings, i.e., *BP* for Δ*T* as obtained from the VFT expression for η (violet) and *B*_s_*P* for Δ*T*_s_ (yellow). The red continuous line results from the analytical value of Δ*T*_HBM_ evaluated from *T*_0_ and *T*_s_ following the prescriptions in ref (27). Results of Δ⟨*T*_av_⟩ at 34 nm and Δ*T_s_* calculated with the Laplace equation are represented as a dashed yellow line for Δ*T*_s_ and as a dotted blue line for Δ⟨*T*_av_⟩. See the main text for details.

The first set of optical trapping and nanothermometry experiments were performed with a commercial optical tweezers setup described in the Experimental Section and schematically represented in Figure 2a. Briefly, a single particle is trapped close to the beam waist of a tightly focused laser beam (λ = 1064 nm). The forward scattered light carries information about the Brownian fluctuations of the particle around the trapping position, and it is collected and guided to a quadrant photodetector (QPD) that provides a voltage signal proportional to such fluctuations. Typically, that signal is analyzed in terms of the power spectral density (PSD), i.e., the Fourier transform of its autocorrelation function.^15^ For an overdamped system where a particle is in equilibrium with a thermal bath at absolute temperature *T* and trapped in a parabolic potential well, the PSD depends on the frequency, *f*, following a Lorentzian function
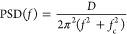
1characterized by the diffusion coefficient of the particle *D* = *k*_B_*T*/γ and the corner frequency of the particle in the trap *f*_c_ = κ/γ, where *k*_B_ is the Boltzmann constant and γ = 6πη*R*_H_ is the Stokes friction coefficient for a sphere with hydrodynamic radius *R*_H_ immersed in a fluid of viscosity η. Finally, *f*_c_ is proportional to the stiffness of the trap, κ, which in turn is proportional to the power of the trapping laser, *P*. Therefore, one typically finds *f*_c_ ∝ *P* in the case of nonabsorbing particles. It should be remarked that experiments with trapped particles were performed at distances larger than 10 μm, ensuring that no corrections to Faxen’s law are needed.^39^

In our case, since the temperature of the liquid is unknown due to light absorption of the NPs, the standard calibration of displacement based on PSD cannot be used.^15^ However, we can evaluate the effects of heating via *f*_c_, which can be determined without calibration since its measurement only depends on the internal clock of the electronic system. Therefore, the PSD of the Brownian fluctuations is fitted to a Lorentzian function, from which *f*_c_ can be evaluated (see Figure 2b). From a classical perspective, the laser power-to-corner frequency *ratio*, *P*/*f*_c_, should be proportional to the solvent viscosity, η(*T*), that depends on the temperature *T* in a nontrivial but well-known way. In the case of nonabsorbing particles, the temperature stays constant, and the same would occur for the ratio *P*/*f*_c_. However, as is clearly seen in Figure 2c, this is not the case in our experiments, where we notice a decrease in the ratio *P*/*f*_c_ as the laser power increases. We interpret this as a reduction of the solvent viscosity due to the rise of the temperature of the trapped particle, which consequently heats up the surrounding liquid. Accepting that the temperature can be linearly related to the laser power in the trap *P* as *T* = *T*_0_ + *BP*,^23,40^ where *T*_0_ is the chamber temperature before laser heating, the experimental data are nonlinearly fitted to the function
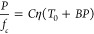
2where *B* and *C* are fitting parameters. The shear viscosity was assume to follow a Vogel–Fulcher–Tammann (VFT) dependence on temperature, i.e,
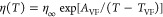
3where the coefficients *A*_VF_, η_*∞*_ and *T*_VF_ are obained from ref (41). After this procedure, experimental results for the classical Brownian temperature are shown in Figure 2d as violet symbols, together with the fitting curve (*R*^2^ = 0.92, χ_ν_^2^ = 0.34). The obtained heating coefficient, *B*, was 368 ± 51 K W^–1^, which is of the same order of magnitude as those previously obtained for different gold nanostructures.^20,42^ With this procedure, a straight line of slope *BP* is expected (see the violet continuous line in Figure 2d). To obtain experimental values of Δ*T* (shown as violet squares in Figure 2d), we applied eq 2 to the experimental value of *P*/*f*_c_. From these values, a temperature increment for the liquid around the particle is obtained that can be as high as Δ*T* ≃ 50 K, as shown in Figure 2d.

However, the meaning of the temperature obtained using eq 2 is not clear, since the trapped particle is hotter than the surrounding liquid, and a gradient of temperature is expected to develop,^27^ which can even be complicated by anomalies in the structure of water that may appear in this temperature range.^43^ The temperature gradient, together with a concomitant viscosity one, affects the Brownian dynamics in the trap, whose evaluation can help us interpret the obtained results in a more reliable way. In this case, a system composed of a particle that is hotter than the environment due to a continuous energy input reaches a nonequilibrium steady state where the surface temperature of the particle *T*_*s*_ gets a constant value that is higher than the surrounding liquid, and a temperature gradient close to it relaxes this value toward that of the thermal bath far from it. The theory of HBM shows that the dynamics of a particle in such a situation can be described by an *effective* diffusion coefficient *D*_HBM_ = *k*_B_*T*_HBM_/6πη_HBM_*R*_H_ given by an effective temperature *T*_HBM_ and an effective viscosity η_HBM_ that would return the measured diffusion coefficient of the very same particle placed in an equilibrium bath with those properties.^27^

We applied the HBM theory to account for the out-of-equilibrium nature of the system by exploiting eq 2 but employing the expression for η_HBM_ from ref (27) instead, which we reproduce here for the sake of completeness
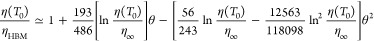
4with θ = (*T*_*s*_ – *T*_0_)/(*T*_0_ – *T*_VF_), *T*_*s*_ being the solvent temperature at the hydrodynamic boundary or surface temperature. Since η_HBM_ incorporates the driving force responsible for the heat flux, θ, *T*_s_ is obtained at each laser power by fitting the data in Figure 2c. In a first approximation, the relation *T*_*s*_ = *T*_0_ + *B*_*s*_*P* should also hold, so this way we obtain the equivalent heating coefficient at the surface, *B*_*s*_ = 830 ± 110 K W^–1^ according to the fitting (*R*^2^ = 0.92, χ_ν_^2^ = 0.41). Similarly, the expected straight line of slope *B*_s_*P* is shown as a continuous yellow line in Figure 2d, while experimental data obtained from the measured *P*/*f*_c_ values are plotted as full yellow triangles. Notably, values of *T*_*s*_ above 100 °C are observed at moderate and high laser powers without any manifestation of cavitation during the experiments. This effect has been previously reported and explained from different perspectives,^23,44,45^ from which the more plausible one under our experimental conditions is the formation of a surface-tension-induced metastable and locally stretched fluid that prevents the formation of nano-/microbubbles.^23^

In a second step, the *effective* HBM temperature can be obtained^27^ using Δ*T*_HBM_ ≃ Δ*T*_*s*_/2 – [1 – ln(η_0_/η_*∞*_)]Δ*T*_*s*_^2^/(24*T*_0_). The data (shown as red circles in Figure 2d) are systematically higher (∼25% at the higher laser power value) than the classical *Brownian* temperature increments derived above. In other words, the effective temperature governing the dynamics of the trapped particle is higher than what is expected from the presumption that the dynamics follow classical Brownian motion, this effect being more apparent as the laser power increases. This happens because of the development of viscosity and temperature gradients within the nanoparticle boundaries and its surroundings. This observation may, at least partly, explain the discrepancies observed for different thermometric methods in ref (23), where the effects derived from the HBM were ignored. The confirmation that, under strong heating conditions, assuming a constant temperature provokes the underestimation of the center-of-mass motion of the particle sets traditional thermometric estimations and interpretations aside when manipulating efficient light-to-heat conversion nanomaterials.

A quick look at the so-obtained values of the temperature increments with *P* allows one to observe that the expected linear trend is not fulfilled above intermediate laser powers. In principle, this outcome may suggest non-Fourier *steady-state* behavior at energy fluxes in the trap of the order of 10^12^ W m^–2^. Monte Carlo simulations of heating nanoparticles have reported positive deviations of the surface temperature from the predictions of the heat transport constitutive equation, which are ascribed to noncontinuous thermal conductivity and a mismatch between the solvent and the solid nanoparticle surface.^46^ Other similar effects have been experimentally observed with trapped nanoparticles, reporting a higher-than-expected variance of the instantaneous velocity of trapped Brownian particles due to a phase change in the water structure.^43^ Negative departure from linearity has also been previously observed in some experimental setups.^47^ However, we are not aware of theoretical treatments for describing these phenomena under the optical trapping conditions. Furthermore, temperature-dependent hydration/dehydration of the polymeric PEG coating^48^ might affect the local mass transport and heat exchange between the solvent layers, and water anomalies also occur within the accessed temperature range.^49^ Whether the captured experimental anomaly is due to either of these contemplated or unreported effects or even to a numerical artifact provoked by the strong variation of the viscosity with the temperature, which is more dramatically reflected in the high-energy regime, is on the table now.

Additional insights into the experimental data can be provided from the analytical temperature profiles that were assessed as follows. The average absorption cross-sections determined above via the discrete dipole scattering calculations are employed to evaluate the absorbed power, which is estimated under the proviso of strong confinement as^50^
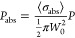
5where *W*_0_ = λ/(πNA) is the waist radius and NA is the numerical aperture of the objective. The computed absorbed power was set as a seed for evaluating the temperature profiles, *T*(*r*), outside the nanoparticle surface, *s*, from the analytical solution of the Fourier equation around an *effective* spherical heating source at rest in a water bath, i.e., the *steady-state* Laplace equation
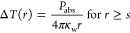
6where κ_w_ = 0.6 W K^–1^ m^–1^ is the thermal conductivity of water and *r* the radial distance from the particle center. Some examples of these profiles are shown in Figure 2e. Since the experimental value of *B* implicitly considers the solvent heating, for comparative purposes this contribution was added *ad hoc* according to the rate *B*_w_ ≈ 12 K W^–1^ obtained in ref (51) for water at 1064 nm. The average temperature increment at a distance *R* = *r* – *s*, Δ⟨*T*_av_(*r*)⟩, is then analytically obtained from the weighted volume integral:
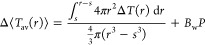
7

Theoretical results obtained from eq 7 are in excellent agreement with the experimental data when Δ⟨*T*_av_⟩ values were obtained at a distance *r* of 34 nm, just 5 nm away of the hydrodynamic diameter, as shown as light blue dotted lines in Figure 2d. One may interpret the radial distance at which the *steady-state* Δ⟨*T*_av_⟩ evaluated from the Laplace equation is equivalent to Δ*T*_HBM_ as the point where the average kinetic energy of the molecules in the solvent bath stops being influenced by the HBM. This distance roughly coincides with the thickness of ∼70 monolayers of water, from which one may deduce the solvent volume required to subdue the strong effect of temperature gradients by thermalization. Moreover, assuming that the generated heat power needed to increase the bath temperature to the effective *T*_HBM_ is wholly absorbed within this solvent volume, we estimated the time required to reach the HBM effective temperature is ∼200 ns.

#### 2.2.2 Emission-Based Nanothermometry

**Figure 3:**
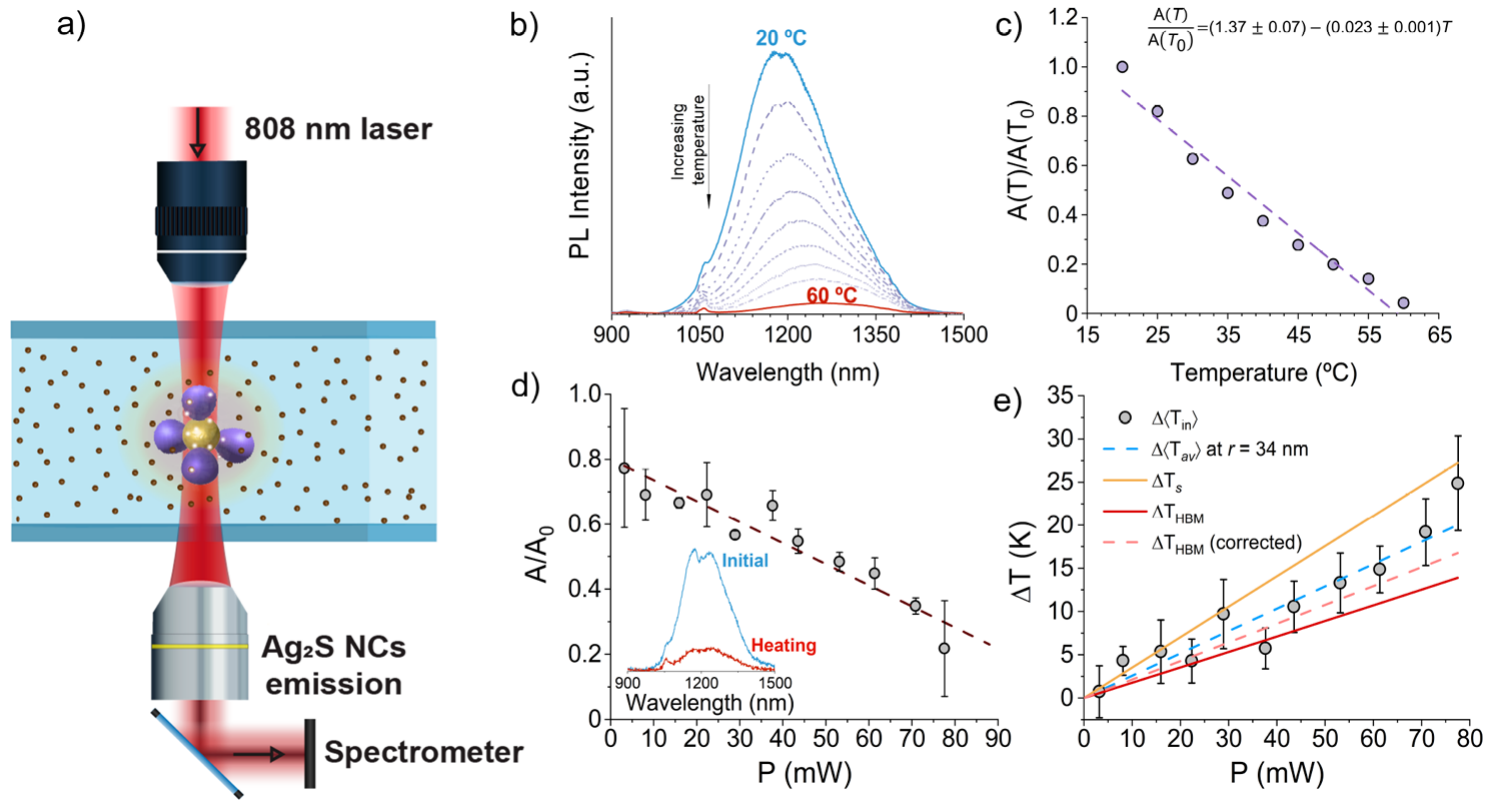
Emission-based nanothermometry. (a) Scheme of the trapping setup for emission-based nanothermometry. (b) Experimental variation of the photoluminescence spectra of an Ag_2_S NC suspension with the temperature. (c) Calibration curve of the experimental normalized photoluminescence spectral area of the Ag_2_S NC suspension versus the externally controlled temperature. Error bars are smaller than the symbol size. The fitting line is included as a dashed line. (d) Normalized photoluminescence spectral area as a function of the laser power (808 nm) as measured in the trapping experiments. The inset shows an example of the raw detected spectra at the lowest (initial) and highest (final) laser powers. Error bars are calculated from the standard deviation of, at least, triplicate experiments. (e) Experimental Δ⟨*T*_in_⟩ temperature obtained from the combination of the experimental data in bulk Ag_2_S NCs suspensions in (c) with the linear calibration in (d) (gray symbols). Error bars are propagated from those of (d) and the determination error of the calibration in (d). Theoretical results obtained for Δ⟨*T*_av_⟩ to a radial distance *r* of 34 nm are also included as a dashed blue line. The continuous yellow line stands for Δ⟨*T*_*s*_⟩ as obtained from eqs 3 and 4. From *T*_*s*_, the HBM temperature is evaluated and included as a red continuous line. Finally, considering the water and nanothermometers heating rate at 808 nm was set at *B*_nt_ = 42 K W^–1^ according to the blank experiments detailed in the Experimental Section, Δ*T*_HBM_ has been corrected with *B*_nt_, the results being shown as a dashed orange line. See the main text for details.

The second set of experiments was accomplished by monitoring the NIR emission of a dispersion of nanothermometers, in which the NFs are embedded. The schematic representation of the trapping and photoluminescence (PL) detection setup is sketched in Figure 3a and detailed in the Experimental Section. In previously published results, it was shown that the intensity of the NIR emission at ∼1200 nm of Ag_2_S nanocrystals (NCs) can act as reversible nanothermometers^52,53^ and are here used for nanothermometry purposes in optical trapping. Briefly, we employed a commercial suspension of Ag_2_S-PEG-COOH nanothermometers that prior to the trapping experiments were calibrated following a procedure in which the temperature dependence of the emission of an NCs dispersion is taken as the transduction signal. In Figure 3b this variation is presented. The changes in the area of the emission band with temperature were used to determine the thermal sensitivity of the Ag_2_S NCs that followed the linear relation A(*T*)/A(*T*_0_) = 1.37_7_ – 0.023_1_*T* (*R*^2^ = 0.96) as shown in Figure 3c, where *A*(*T*)/*A*(*T*_0_) is the *ratio* of the spectral area at temperature *T*, *A*(*T*), and that at a reference temperature *T*_0_, *A*(*T*_0_). From this calibration, it can be concluded that using Ag_2_S NCs for nanothermometry is restricted to temperatures up to ∼60 °C. Then, an aqueous colloidal suspension of NPs and Ag_2_S NCs was placed in a homemade single-beam optical trapping setup, described in the Experimental Section, which uses an 808 nm linearly polarized single mode fiber-coupled laser diode. The laser was used for both trapping the nanoflowers and exciting the NCs in their closer surroundings. It should be noted that the presence of a nanoflower in the optical trap diminishes the NC emission by about ∼20%, as extrapolated from the calibration line in Figure 3d at *P* → 0. The NC emission was monitored with a NIR spectrometer fiber coupled to the system. The normalized photoluminescence area of the NC dispersion was measured as a function of the laser power (808 nm) in the trapping setup as shown in Figure 3d. The inset shows an example of the raw detected spectra at the lowest and highest laser powers. Finally, the spectral data were converted to temperature data using the calibration line obtained for the nanothermometer suspension. The final results are collected in Figure 3e as full symbols.

It must be stressed that our measurement through the emission changes in the NIR spectra upon heating reflects the average internal temperature of the Ag_2_S nanocrystals contained in a liquid volume around the trapped particle, ⟨*T*_in_⟩, and this is not a direct observable evincing the trapped nanoparticle dynamics or the surface temperature from which *T*_HBM_ can be derived. Particularly, the experimental values of Δ⟨*T*_in_⟩ reflect real differences in the average temperature around the heated nanoparticle due to either the NC or NF absorption. As shown as a dashed blue line in Figure 3e, we found that the values of Δ⟨*T*_av_(*r*)⟩ obtained from the Laplace equation at *r* = 34 nm, including the experimental heating contribution of the solvent and the nanothermometers, *B*_nt_ (obtained from external calibration, see Experimental Section), are in excellent agreement with the Δ⟨*T*_in_⟩ data. Therefore, the question that arises after these results is whether the radial distance required to obtain an average temperature compatible with the spectroscopic data is representative of the effective solvent boundary affected by the HBM. Although the overall agreement between the experimental Δ⟨*T*_in_⟩ data and the theoretical values of Δ⟨*T*_av_(*r*)⟩ at a radial distance of 34 nm is consistent with the experiments at 1064 nm, solving this fundamental issue is cumbersome from any angle. In the next section, we try to address this issue directly by comparing both data sets.

#### 2.2.3 Comparison of the Two Data Sets and Discussion

In this section, we try to find a connection between the two types of performed measurements. Some hints can be provided by pondering that in a first approximation both experiments should be fully equivalent in terms of heat generation if the absorbed power is considered a common variable, regardless of the trapping wavelength. Provided the nice agreement found in the experiments performed at 1064 nm, we evaluated the surface temperature from the Laplace equation employing the ⟨σ_abs_⟩ value at 808 nm obtained via DDSCAT calculations, from which *T*_HBM_ can be derived. The analytical results of both temperatures are plotted as continuous (yellow for Δ*T*_*s*_ and red for Δ*T*_HBM_) lines in Figure 3e. There is a nice agreement between Δ⟨*T*_av_(*r*)⟩ and Δ*T*_HBM_, singularly considering that no contributions of the solvent or nanothermometers are contemplated in the HBM temperature. Under the naive assumption that this contribution *B*_nt_ is additive to the theoretical value of Δ*T*_HBM_, we obtained the dashed orange line in Figure 3e (denoted as Δ*T*_HBM_ corrected), which is in even closer agreement with Δ⟨*T*_av_(*r*)⟩. This is a manifestation that Δ⟨*T*_av_(*r*)⟩ is closely related with Δ*T*_HBM_ as evaluated from the trapped particle heating once the contribution of its surroundings is accounted for by, in a first approximation, simple addition.

**Figure 4:**
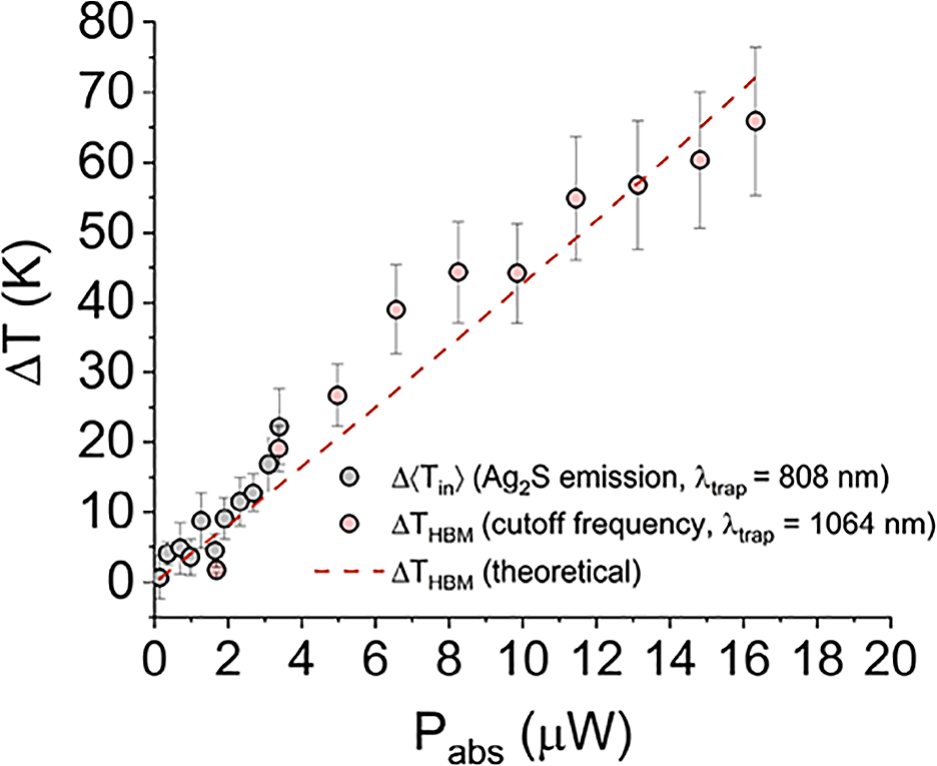
Comparison between the temperatures obtained from the two methods. Δ*T*_HBM_ increments obtained with the corner frequency (light red symbols) and Δ⟨*T*_in_⟩ as obtained with the emission of Ag_2_S NCs (gray symbols). The continuous red line represents the theoretical calculation of Δ*T*_HBM_ derived from the HBM theory assuming a temperature profile as defined in eq 6.

Following this reasoning, the plot of the values of Δ*T* coming from both sets of experiments against *P*_abs_ should overlap if the extracted Δ*T* values have some physical resemblance between them. Note that *P*_abs_ is evaluated according to eq 5 for each experimental setup, i.e., NA, λ, and σ_abs_. In other words, we can disclose if Δ⟨*T*_in_⟩ is comparable with Δ*T*_HBM_ in the whole power regime and, consequently, whether remote-nontrapped thermometers are reliable tools to described the HBM dynamics within their thermal working range. This comparison is presented in Figure 4, where the theoretical value of Δ_HBM_ is also included. Note that one can evaluate the theoretical Δ*T*_*s*_ value from the Laplace equation independently of the concrete initial temperature *T*_0_ but, since *T*_HBM_ is a function of *T*_0_ and our experimental values are different in ∼2 K in each setup, the continuous line representing the theoretical Δ*T*_HBM_ value has been calculated for the average *T*_0_. The agreement between experimental and theoretical data is such that the RMSE is ∼6 K. In addition, the contribution of the nanothermometers has been subtracted from the experiments at 808 nm for the sake of comparison. The representation of those sets of data in the absorbed power range of 0–20 μW indicates that the values of Δ⟨*T*_in_⟩ derived from auxiliary aide-emissive nanothermometers nicely correlate, within experimental uncertainties, with those defining the particle dynamics in the framework of the HBM theory when the laser power was appropriately scaled with the absorption cross-section of the nanoparticles. This result indicates both negligible effects of the nanothermometers on the physical properties of the heat source and *vice versa*, and that the Fourier constitutive equation remains applicable in the low-fluence regime at the nanoscale, even for estimating the HBM temperature.

Finally, we must point out that given the large temperature increase we measured, it could be expected that convection should be driven in the fluid around the trapped particles, affecting both the temperature gradients around particles and their dynamics. However, no signatures of convection were observed in our experiments, which could have been seen as forced motion of nearby particles or deviations from the low-frequency plateau in the PSD. This is in agreement with previous works, where very small velocities were expected to occur for individual plasmonic nanoparticles,^54^ but either collective effects^55^ or the combination with electric fields is needed in order to force significant convection.^56,57^ In fact, previous experiments involving the trapping of plasmonic nanoparticles that reported the motion of nearby particles were explained in terms of thermophoresis and did not involve the occurrence of convection.^58^

## 3 Conclusions

To sum up, the temperature and heat generation by absorbing anisotropic hybrid nanostructures under optical trapping conditions were experimentally and theoretically achieved on the basis of the following

(i) *The evaluation of the balance of the optical forces on the confining trap as a function of the laser power*. The experimental temperature data were extracted from the expected dependence of the power-to-corner frequency *ratio* with the *effective* medium viscosity, as established in the HBM theory. The order of magnitude of the obtained heating parameter in the classical Brownian approximation is in agreement with previously reported data. In contrast, the HBM method led to temperature increments systematically higher than those obtained from the classical Brownian motion theory for a particle immersed in a thermal bath. This fact points to the necessity of applying the sparingly employed HBM theory to extract valid information from thermometric measurements when handling highly absorbing particles and questions the traditional meaning of *temperature* as far as single-particle nanothermometry is concerned. It was also verified that at low energy fluxes, the Fourier equation remains applicable to obtain the HBM *temperature* from the directly estimated value of *T*_*s*_.

(ii) *The calibration of a colloidal suspension of NIR emissive nanothermometers embedding the trapped nanoparticles*. Average internal temperature increments within the trapping volume have been accessed directly by the near-infrared emission of Ag_2_S NCs acting as NIR nanothermometers. The values obtained within this approach constitute a sort of average temperature, and it was found that it might be resembling the HBM temperature after correcting the heating contribution of the environment. This asseveration was demonstrated by proving that by merging the experimental data coming from both sets of results for the dependence of Δ*T* with *P*, obtained in two different laboratories and with two different trapping setups operating at different trapping wavelengths, they collapsed when plotted against the absorbed power.

These findings represent a robust viewpoint of the temperature measurements in single-particle systems in relation to the experimental route from which the heating properties are derived. That said, while experimental pieces of evidence indicate that the internal temperature determined from the appropriate calibration of NIR-emissive nanothermometers capture that dictating the particle dynamics (i.e., the HBM temperature), no rigorous theoretical validation of such conclusion has been reported to date. Further applications of the protocol initiated in this work might yield a reinterpretation or validation of the trapping experiments on self-emissive nanothermometers.^59^ On a final note, in light of the importance of the HBM theory to elucidate nanothermometric data coming from optical trapping setups, it seems urgent to generalize it to incorporate the local heating contribution of both the nearby particles and solvent and the effects of the particle softness^40^ into the formalism.

## 4 Experimental Section

### 4.1 Chemical Reagents

Gold(III) acetate, iron(III) chloride, sodium oleate, oleic acid 99%, oleylamine, 1,2-hexadecanediol, gallic acid, polyethylene glycol (PEG) 3000 Da, 1-octadecene, triethylamine, 4-dimethylaminopyridine (DMAP), and ethanol 99% were obtained from Sigma-Aldrich. Dimethyl sulfoxide (DMSO), toluene, acetone, hexane, chloroform, dichloromethane (DCM), and tetrahydrofuran (THF) were supplied by Acros Organics. All reagents were used as received without further purification. Milli-Q water (18.2 MΩ cm) was obtained from a Millipore system. Ag_2_S NCs were obtained from NIR Optics Technology (average size ∼9 nm).

### 4.2 Seed-Growth Synthesis of Au@Fe_**3**_O_**4**_ Nanoflowers

#### 4.2.1 Synthesis of Iron(III) Oleate

The synthesis was done following ref (60). Briefly, a mixture of 10.8 g of iron(III) chloride (40 mmol) and 36.5 g of sodium oleate (120 mmol) was dissolved in a solvent mixture with 80 mL of ethanol, 60 mL of distilled water, and 140 mL of hexane. The resulting solution was heated to 60 °C for 4 h in hexane reflux under an inert atmosphere. The reaction mixture was then cooled to room temperature. The organic phase was washed 3 times with distilled water, and the hexane was evaporated in a rotavap.

#### 4.2.2 Synthesis of the Gold Seeds

The synthesis was done following ref (12) with some modifications. 50 mg of gold(III) acetate was dissolved in a mixture of 0.8 mL of oleic acid, 0.6 mL of oleylamine, 100 mg of 1,2-hexadecanodiol, and 5 mL of 1-octadecene. The mixture was heated under vacuum to 120 °C with a heating rate of 5 °C min^–1^ and kept at this temperature for 30 min. After cooling down, it was washed twice with 36 mL of an ethanol/acetone mixture (1:1, v:v) and centrifuged at 8000 rpm for 20 min. Then, the gold NPs were dispersed in 10 mL of hexane.

#### 4.2.3 Synthesis of Au–Fe_3_O_4_ Nanoflowers

The synthesis was conducted as previously described by some of us.^31^ 1 mL of Au NPs was mixed with 0.63 mL of oleylamine, 0.66 mL of oleic acid, 0.645 mL of 1,2-hexadecanodiol, 10 mL of 1-octadecene, and 0.125 g of iron(III) oleate. The mixture was heated under vacuum to 120 °C and kept under this condition for 20 min. Then, the temperature was raised to 200 °C and the mixture kept at this temperature for 120 min. The temperature was raised again to 315 °C with a heating rate of 5 °C min^–1^ and the mixture kept at this temperature for 30 min (growth of iron). After cooling, it was washed with a mixture of ethanol/acetone (1:1) and centrifuged. This step was done twice. Then, the gold NPs were dispersed in 10 mL of hexane.

### 4.3 Functionalization of the Au–Fe Nanoflowers

The synthesis of the appropriate PEGylated ligand and the subsequent ligand exchange process were conducted as described previously by some of us in ref (61). In the following, we provide the most important information.

#### 4.3.1 Synthesis of the PEGylated Ligand

First, we proceeded with a dropwise addition of DCC (1 g in 5 mL of THF) to a solution of 3 g of PEG, 170 mg of gallic acid, and 24 mg of DMAP in 100 mL of THF and 10 mL of DCM. The resulting mixture was stirred overnight at room temperature, and finally, it was filtered through a filter paper and the solvents were rotavaporated.

#### 4.3.2 Ligand Exchange

The ligand exchange procedure was performed following ref (61). In short, in a glass vial was placed a solution containing 1 mL of NPs (10 g/L of Fe), 1 mL of gallol-PEG_*n*_-OH in a concentration of 0.1 M in CHCl_3_, and 50 μL of triethylamine. The mixture was ultrasonicated for 1 h and kept for 4 h at 50 °C. At this point, it was diluted with 5 mL of toluene, 5 mL of Milli-Q water, and 10 mL of acetone. Then, it was shaken and the nanoparticles were transferred to the aqueous phase. After that, the aqueous phase was collected in a round-bottom flask and the residual organic solvents were rotavaporated. Then, the gallol-derived NPs were purified in centrifuge filters with a molecular weight cutoff of 100 kDa at 450 rcf. In each centrifugation, the functionalized NPs were resuspended with Milli-Q water. The purification step was repeated several times until the filtered solution was clear. After the purification, the gallol-derived NPs were resuspended in PBS buffer. Finally, to improve the monodispersity and remove aggregates, this solution was centrifuged at 150 rcf for 5 min and it was placed onto a permanent magnet (0.6 T) for 5 min.

### 4.4 Physicochemical Characterization of the Au–Fe Nanoflowers

The size distribution was obtained by TEM imaging a carbon-coated copper grid in which a sample suspension of ∼1 g/L (Fe + Au) was deposited dropwise. The images were acquired on an FEI Tecnai G2 Twin microscope working at 100 kV. Size histograms were then calculated by averaging the characteristic dimensions of 100 nanoparticles with the aid of the free ImageJ software. The extinction spectra were recorded on a Jenway Series 67 spectrophotometer. The measurements were performed in a very dilute suspension to avoid potential collective effects affecting the scattering contribution due to the potential aggregation of the nanoparticles.

### 4.5 Piriform Curve for Particle Modeling

The geometry used to implement the simulations is based on the revolution of the piriform curve, which depends on only the value of a parameter β and is given in parametric form by the expression
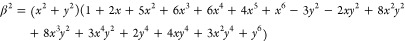
8

### 4.6 Optical Trapping at 1064 nm

The experiments at 1064 nm were performed in a NanoTracker-II optical tweezers (JPK-Bruker) device. In this setup, the infrared laser was tightly focused by a high numerical aperture objective (63 × , NA_*b*_ = 1.2) to a diffraction-limited spot where trapping occurs. The forward-scattered light is collected by a second objective and guided to a QPD instrument to record the X-Y traces that generate a voltage signal sampled at a frequency of 50 kHz. This voltage is proportional to the displacement of the particles inside the optical trap. Spurious bright flashes in the video were assumed to be a consequence of a multiple trapping event and, in such cases, the PSD and the corresponding *f*_c_ were discarded.

### 4.7 Optical Trapping at 808 nm

The optical trapping experiments were performed in a homemade single-beam optical trapping setup. Drops of aqueous suspensions of MCNPs and NCs, previously stirred to avoid clusters, were pipetted into a 120 μm height and 13 mm diameter microchamber that was placed in the optical tweezer setup. A linearly polarized 808 nm single-mode fiber-coupled laser diode was focused into the chamber containing the sample by using an LCPLN 100× IR Olympus microscope objective with a numerical aperture of 0.85 that led to a spot size of ∼0.63 μm. The tightly focused laser beam was used for both trapping the nanoflowers and exciting the NCs in their closer surroundings. Real-time optical imaging of the NCs was achieved by coupling a white LED, focused on the sample by a 40× objective lens, and using a CMOS camera incorporated into the system. The lower objective lens was used as a light condenser but also served as a collector lens to focus the NCs emission into an OceanInsight NIR spectrometer, fiber-coupled to the system.

### 4.8 External and Internal Ag_**2**_S NC Calibration

For the external calibration of the Ag_2_S in bulk dispersions, the temperature of the sample was controlled by a Linkam PE120 stage (±0.1 °C). The NC dispersion was excited with a fiber-coupled 808 nm single-mode diode laser, while the NIR emission spectra were collected with an OceanInsight high-performance NIR spectrometer (900–1700 nm). A high-quality long-pass filter (750 nm) was placed in the detection path so that the noise level registered by the camera did not exceed 0.5% of the signal generated by the Ag_2_S NCs. While the heating contribution of water in the trap might be set to *B*_w_ = 3 K W^–1^,^62^ the contribution of the Ag_2_S NCs is unknown. To evaluate their contribution, a set of experiments on the colloidal suspension under trapping conditions (but without trapped MCNPs) were performed. The temperature increments were evaluated from the internal calibration curve, and the solvent plus NC heating coefficient, *B*_nt_, was determined from the slope of Δ*T* against laser power measurements. The calibration curve is shown in Figure S4. The outcome of these experiments was *B*_nt_ ≈ 42 K W^–1^.

## Supporting Information Available

Size distribution of the MCNPs as obtained from electronic microscopy, geometrical models for the petals employed in the calculation of the scattering profiles, example of a 3D plot of the calculated electric field intensity for the model T3 at 1064 nm, and calibration curve for evaluating the nanothermometer contribution to the observed temperature in the emission-based method (PDF)

## References

[ref1] Caro C.; Gámez F.; Quaresma P.; Páez-Muñoz J. M.; Domínguez A.; Pearson J. R.; Pernía-Leal M.; Beltrán A. M.; Fernandez-Afonso Y.; la Fuente J. M. D.; Franco R.; Pereira E.; García-Martín M. L. Fe_3_O_4_-Au Core-Shell Nanoparticles as a Multimodal Platform for *in Vivo* Imaging and Focused Photothermal Therapy. Pharmaceutics 2021, 13, 416 10.3390/pharmaceutics13030416. 33804636 PMC8003746

[ref2] Ximendes E.; et al. Infrared-Emitting Multimodal Nanostructures for Controlled *in vivo* Magnetic Hyperthermia. Adv. Mater. 2021, 33, 2100077 10.1002/adma.202100077. PMC11468761 34117667

[ref3] Ha M.; Kim J.-H.; You M.; Li Q.; Fan C.; Nam J.-M. Multicomponent Plasmonic Nanoparticles: From Heterostructured Nanoparticles to Colloidal Composite Nanostructures. Chem. Rev. 2019, 119, 12208–12278. 10.1021/acs.chemrev.9b00234. 31794202

[ref4] Lázaro M.; Lupiáñez P.; Arias J. L.; Carrasco-Jiménez M. P.; Delgado Á. V.; Iglesias G. R. Combined Magnetic Hyperthermia and Photothermia with Polyelectrolyte/Gold-Coated Magnetic Nanorods. Polymers 2022, 14, 4913 10.3390/polym14224913. 36433039 PMC9693010

[ref5] Díez A.; Rincón-Iglesias M.; Lanceros-Méndez S.; Reguera J.; Lizundia E. Multicomponent magnetic nanoparticle engineering: the role of structure-property relationship in advanced applications. Mater. Today Chem. 2022, 26, 101220 10.1016/j.mtchem.2022.101220.

[ref6] Yankeelov T.; Abramson R.; Quarles C. Quantitative multimodality imaging in cancer research and therapy. Nature Reviews Clinical Oncology 2014, 11, 670–680. 10.1038/nrclinonc.2014.134. PMC4909117 25113842

[ref7] Mokhtari R.; Homayouni T.; Baluch N.; Morgatskaya E.; Kumar S.; Das B.; Yeger H. Combination therapy in combating cancer. Oncotarget 2017, 8, 38022–38043. 10.18632/oncotarget.16723. 28410237 PMC5514969

[ref8] Nguyen T. T.; Mammeri F.; Ammar S. Iron Oxide and Gold Based Magneto-Plasmonic Nanostructures for Medical Applications: A Review. Nanomaterials 2018, 8, 149 10.3390/nano8030149. 29518969 PMC5869640

[ref9] Ding Q.; Liu D.; Guo D.; Yang F.; Pang X.; Che R.; Zhou N.; Xie J.; Sun J.; Huang Z.; Gu N. Shape-controlled fabrication of magnetite silver hybrid nanoparticles with high performance magnetic hyperthermia. Biomaterials 2017, 124, 35–46. 10.1016/j.biomaterials.2017.01.043. 28187393

[ref10] Zhang W.; et al. A Nanoscale Shape-Discovery Framework Supporting Systematic Investigations of Shape-Dependent Biological Effects and Immunomodulation. ACS Nano 2022, 16, 1547–1559. 10.1021/acsnano.1c10074. 34958549 PMC8793145

[ref11] Baffou G.; Quidant R. Thermo-plasmonics: using metallic nanostructures as nano-sources of heat. Laser & Photonics Reviews 2013, 7, 171–187. 10.1002/lpor.201200003.

[ref12] Tancredi P.; da Costa L. S.; Calderon S.; Moscoso-Londoño O.; Socolovsky L. M.; Ferreira P. J.; Muraca D.; Zancheta D.; Knobel M. Exploring the synthesis conditions to control the morphology of gold–iron oxide heterostructures. Nanoresearch 2019, 12, 1781–1788. 10.1007/s12274-019-2431-7.

[ref13] Zhang R.; Cheng K.; Antaris A.; Ma X.; Yang M.; Ramakrishnan S.; Liu G.; Lu A.; Dai H.; Tian M.; Cheng Z. Hybrid anisotropic nanostructures for dual-modal cancer imaging and image-guided chemo-thermo therapies. Biomaterials 2016, 103, 265–277. 10.1016/j.biomaterials.2016.06.063. 27394161 PMC4970737

[ref14] Tarkistani M. A. M.; Komalla V.; Kayser V. Recent Advances in the Use of Iron–Gold Hybrid Nanoparticles for Biomedical Applications. Nanomaterials 2021, 11, 1227 10.3390/nano11051227. 34066549 PMC8148580

[ref15] Gieseler J.; Gomez-Solano J. R.; Magazzù A.; Castillo I. P.; García L. P.; Gironella-Torrent M.; Viader-Godoy X.; Ritort F.; Pesce G.; Arzola A. V.; et al. Optical tweezers-from calibration to applications: a tutorial. Advances in Optics and Photonics 2021, 13, 74–241. 10.1364/AOP.394888.

[ref16] Hajizadeh F.; S Reihani S. S. Optimized optical trapping of gold nanoparticles. Opt. Express 2010, 18, 551–559. 10.1364/OE.18.000551. 20173874

[ref17] Kang P.; Serey X.; Chen Y.-F.; Erickson D. Angular Orientation of Nanorods Using Nanophotonic Tweezers. Nano Lett. 2012, 12, 6400–6407. 10.1021/nl303747n. 23145817

[ref18] Ramachandran N.; Vyas R. N.; Nandheesh D. T.; Yakesh V.; Sebastin R. N. M. Review on optical tweezers in multi-fields. AIP Conf. Proc. 2022, 2527, 030016 10.1063/5.0108108.

[ref19] Odebo Lank N.; Johansson P.; Kall M. Optical tweezing and photothermal properties of resonant dielectric and metallic nanospheres. ACS Photonics 2020, 7, 2405–2412. 10.1021/acsphotonics.0c00292.

[ref20] Andres-Arroyo A.; Wang F.; Toe W.; Reece P. Intrinsic heating in optically trapped Au nanoparticles measured by dark-field spectroscopy. Biomed Opt Express 2015, 6, 3646–3654. 10.1364/BOE.6.003646. 26417530 PMC4574686

[ref21] Andren D.; Shao L.; Odebo Lank N.; Acimovic S. S.; Johansson P.; Kall M. Probing Photothermal Effects on Optically Trapped Gold Nanorods by Simultaneous Plasmon Spectroscopy and Brownian Dynamics Analysis. ACS Nano 2017, 11, 10053–10061. 10.1021/acsnano.7b04302. 28872830

[ref22] Šiler M.; Ježek J.; Jákl P.; Pilát Z.; Zemánek P. Direct measurement of the temperature profile close to an optically trapped absorbing particle. Opt. Lett. 2016, 41, 870–873. 10.1364/OL.41.000870. 26974067

[ref23] Rodríguez-Sevilla P.; Arita Y.; Liu X.; Jaque D.; Dholakia K. The Temperature of an Optically Trapped, Rotating Microparticle. ACS Photonics 2018, 5, 3772–3778. 10.1021/acsphotonics.8b00822.

[ref24] Hajizadeh F.; Shao L.; Andrén D.; Johansson P.; Rubinsztein-Dunlop H.; Käll M. Brownian fluctuations of an optically rotated nanorod. Optica 2017, 4, 746–751. 10.1364/OPTICA.4.000746.

[ref25] Karpinski P.; Jones S.; Šípová Jungová H.; Verre R.; Käll M. Optical Rotation and Thermometry of Laser Tweezed Silicon Nanorods. Nano Lett. 2020, 20, 6494–6501. 10.1021/acs.nanolett.0c02240. 32787173 PMC7496737

[ref26] Rodriguez-Sevilla P.; Zhang Y.; Haro-Gonzalez P.; Sanz-Rodriguez F.; Jaque F.; Sole J. G.; Liu X.; Jaque D. Thermal Scanning at the Cellular Level by an Optically Trapped Upconverting Fluorescent Particle. Adv. Mater. 2016, 28, 2421–2426. 10.1002/adma.201505020. 26821941

[ref27] Rings D.; Schachoff R.; Selmke M.; Cichos F.; Kroy K. Hot brownian motion. Phys. Rev. Lett. 2010, 105, 090604 10.1103/PhysRevLett.105.090604. 20868149

[ref28] Millen J.; Deesuwan T.; Barker P.; Anders J. Nanoscale temperature measurements using non-equilibrium Brownian dynamics of a levitated nanosphere. Nat. Nanotechnol. 2014, 9, 425–429. 10.1038/nnano.2014.82. 24793558

[ref29] Rivière F.; de Guillebon T.; Raynal D.; Schmidt M.; Lauret J. S.; Roch J. F.; Rondin L. Hot Brownian motion of optically levitated nanodiamonds. ACS photonics 2022, 9, 420–425. 10.1021/acsphotonics.1c01642.

[ref30] Jiang Q.; Rogez B.; Claude J.-B.; Baffou G.; Wenger J. Hot Brownian Motion. ACS Photonics 2019, 6, 1763–1773. 10.1021/acsphotonics.9b00519.

[ref31] Christou E.; Pearson J.; Beltrán A.; Fernández-Afonso Y.; Gutiérrez L.; de la Fuente J. M.; Gámez F.; García-Martín M.; Caro C. Iron-Gold Nanoflowers: A Promising Tool for Multimodal Imaging and Hyperthermia Therapy. Pharmaceutics 2022, 14, 636 10.3390/pharmaceutics14030636. 35336012 PMC8955043

[ref32] Campbell J.; Burkitt S.; Dong N.; Zavaleta C. In Nanoparticles for Biomedical Applications; Elsevier: 2020; Chapter 9, pp 129–144.

[ref33] Tang J.; Myers M.; Bosnick K. A.; Brus L. E. Magnetite Fe_3_O_4_ Nanocrystals: Spectroscopic Observation of Aqueous Oxidation Kinetics. J. Phys. Chem. B 2003, 107, 7501–7506. 10.1021/jp027048e.

[ref34] Hemmer E.; Benayas A.; Légaré F.; Vetrone F. Exploiting the biological windows: current perspectives on fluorescent bioprobes emitting above 1000 nm. Nanoscale Horiz 2016, 1, 168–184. 10.1039/C5NH00073D. 32260620

[ref35] Li X.; Lovell J.; Yoon J.; Chen X. Clinical development and potential of photothermal and photodynamic therapies for cancer. Nat. Rev. Clin. Oncol. 2020, 17, 657–674. 10.1038/s41571-020-0410-2. 32699309

[ref36] Chatterjee H. GitHub. https://github.com/ItsHirak/CappedNP.giwebt, Accessed: 2023-07-07.

[ref37] Draine B. T.; Flatau P. J. Discrete-Dipole Approximation For Scattering Calculations. J. Opt. Soc. Am. A 1994, 11, 1491–1499. 10.1364/JOSAA.11.001491.

[ref38] Bohren C. F.; Huffman D. R. Absorption and scattering of light by small particles; Wiley: 2008.

[ref39] Schäffer E.; Nørrelykke S. F.; Howard J. Surface forces and drag coefficients of microspheres near a plane surface measured with optical tweezers. Langmuir 2007, 23, 3654–3665. 10.1021/la0622368. 17326669

[ref40] Fernandez-Rodriguez M. A.; Orozco-Barrera S.; Sun W.; Gámez F.; Caro C.; García-Martín M. L.; Rica R. A. Hot Brownian Motion of Thermoresponsive Microgels in Optical Tweezers Shows Discontinuous Volume Phase Transition and Bistability. Small 2023, 19, 2301653 10.1002/smll.202301653. 37158287

[ref41] Dortmund data bank. www.ddbst.cowebm, Accessed: 2023-07-07.

[ref42] Seol Y.; Carpenter A. E.; Perkins T. Gold nanoparticles: enhanced optical trapping and sensitivity coupled with significant heating. Opt. Lett. 2006, 31, 2429–2431. 10.1364/OL.31.002429. 16880845

[ref43] Lu D.; Labrador-Paez L.; Ortiz-Rivero E.; Frades P.; Antoniak M. A.; Wawrzynczyk D.; Nyk M.; Brites C. D. S.; Carlos L. D.; Garcıa Sole J. A.; Haro-Gonzalez P.; Jaque D. Exploring Single-Nanoparticle Dynamics at High Temperature by Optical Tweezers. Nano Lett. 2020, 20, 8024–8031. 10.1021/acs.nanolett.0c02936. 32936661

[ref44] Baffou G.; Polleux J.; Rigneault H.; Monneret S. Super-Heating and Micro-Bubble Generation around Plasmonic Nanoparticles under cw Illumination. J. Phys. Chem. C 2014, 118, 4890–4898. 10.1021/jp411519k.

[ref45] Kyrsting A.; Bendix P. M.; Stamou D. G.; Oddershede L. B. Heat Profiling of Three-Dimensionally Optically Trapped Gold Nanoparticles using Vesicle Cargo Release. NanoLett. 2011, 11, 888–892. 10.1021/nl104280c. 21188965

[ref46] Merabia S.; Shenogin S.; Joly L.; Barrat J.-L.; et al. Heat transfer from nanoparticles: A corresponding state analysis. Proc. Natl. Acad. Sci. U.S.A. 2009, 106, 15113–15118. 10.1073/pnas.0901372106. 19571000 PMC2741214

[ref47] Sahoo N.; Ghosh S.; Narasimhan A.; Das S. K. Investigation of non-Fourier effects in bio-tissues during laser assisted photothermal therapy. Int. J. Therm. Sci. 2014, 76, 208–220. 10.1016/j.ijthermalsci.2013.08.014.

[ref48] Mallamace F.; Corsaro C.; Stanley H. E. Temperature-Dependent Hydration/Dehydration Behavior of Poly(ethylene oxide)s in Aqueous Solution. Macromolecules 2013, 46, 1956–1961. 10.1021/ma3026282.

[ref49] Shikata T.; Okuzono M.; Sugimoto N. A singular thermodynamically consistent temperature at the origin of the anomalous behavior of liquid water. Sci. Rep 2012, 2, 993 10.1038/srep00993. 23251779 PMC3524791

[ref50] Saleh B. E. A.; Teich M. C. Fundamentals of Photonics, 3rd ed.; Wiley: 2007.

[ref51] Peterman E. J. G.; Gittes F.; Schmidt C. F. Laser-Induced Heating in Optical Traps. Biophys. J. 2003, 84, 1308–1316. 10.1016/S0006-3495(03)74946-7. 12547811 PMC1302707

[ref52] Shen Y.; Harrison H. D. A.; Ximendes E. C.; Lifante J.; Sanz-Portilla A.; Monge L.; Fernandez N.; Chaves-Coira I.; Jacinto C.; Brites C.; Carlos L.; Benayas A.; de la Cruz M. I.; Jaque D. Ag_2_S Nanoheaters with Multiparameter Sensing for Reliable Thermal Feedback during In Vivo Tumor Therapy. Adv. Funct. Mater. 2020, 30, 2002730 10.1002/adfm.202002730.

[ref53] Shen Y.; Lifante J.; Zabala-Gutierrez I.; de la Fuente-Fernández M.; Granado M.; Fernández N.; Rubio-Retama J.; Jaque D.; Marin R.; Ximendes E.; Benayas A. Reliable and Remote Monitoring of Absolute Temperature during Liver Inflammation via Luminescence-Lifetime-Based Nanothermometry. Adv. Mater. 2022, 34, 2107764 10.1002/adma.202107764. 34826883

[ref54] Donner J. S.; Baffou G.; McCloskey D.; Quidant R. Plasmon-assisted optofluidics. Acs nano 2011, 5, 5457–5462. 10.1021/nn200590u. 21657203

[ref55] Ciraulo B.; Garcia-Guirado J.; de Miguel I.; Ortega Arroyo J.; Quidant R. Long-range optofluidic control with plasmon heating. Nat. Commun. 2021, 12, 2001 10.1038/s41467-021-22280-3. 33790293 PMC8012589

[ref56] Ndukaife J. C.; Kildishev A. V.; Nnanna A. G. A.; Shalaev V. M.; Wereley S. T.; Boltasseva A. Long-range and rapid transport of individual nano-objects by a hybrid electrothermoplasmonic nanotweezer. Nature Nanotechnol. 2016, 11, 53–59. 10.1038/nnano.2015.248. 26524398

[ref57] González-Gómez C. D.; Rica R. A.; Ruiz-Reina E. Electrothermoplasmonic flow in gold nanoparticles suspensions: Nonlinear dependence of flow velocity on aggregate concentration. J. Colloid Interface Sci. 2023, 648, 397 10.1016/j.jcis.2023.05.198. 37302223

[ref58] Setoura K.; Tsuji T.; Ito S.; Kawano S.; Miyasaka H. Opto-thermophoretic separation and trapping of plasmonic nanoparticles. Nanoscale 2019, 11, 21093–21102. 10.1039/C9NR05052C. 31402358

[ref59] Labrador-Páez L.; Haro-González P. Luminescent Thermometry: Applications and Uses; Springer: 2023; pp 315–329.

[ref60] Pernia Leal M.; Caro C.; Garcia-Martin M. L. Shedding light on zwitterionic magnetic nanoparticles: limitations for in vivo applications. Nanoscale 2017, 9, 8176–8184. 10.1039/C7NR01607G. 28581000

[ref61] Pozo-Torres E.; Caro C.; Avasthi A.; Paez-Munoz J. M.; Garcia-Martin M. L.; Fernandez I.; Pernia Leal M. Clickable iron oxide NPs based on catechol derived ligands: synthesis and characterization. Soft Matter 2020, 16, 3257–3266. 10.1039/C9SM02512J. 32163076

[ref62] Catala F.; Marsa F.; Montes-Usategui M.; Farre A.; Martin-Badosa E. Influence of experimental parameters on the laser heating of an optical trap. Sci. Rep. 2017, 7, 16052 10.1038/s41598-017-15904-6. 29167481 PMC5700206

