# Supplementary material for: Light-to-Heat Conversion of Optically Trapped Hot Brownian Particles
Source: ACS Nano. 2023 Dec 4;17(24):24961–71. doi: 10.1021/acsnano.3c07086 (PMC10754033; doi:10.1021/acsnano.3c07086)
Supplement: Supplementary file 1 — nn3c07086_si_001.pdf [file nn3c07086_si_001.pdf]

Supporting Information for:
“Light-to-heat conversion of optically trapped hot Brownian particles.

Elisa Ortiz-Rivero[1,†], Sergio Orozco-Barrera[2,†], Hirak Chatterjee[2], Carlos D. González-Gómez[2,3], Carlos Caro[4,5], María-Luisa García-Martín[4,5,6], Patricia Haro González[1], Raúl A. Rica[2,*], Francisco Gámez[7,*].

[1] Nanomaterials for Bioimaging Group, Departamento de Física de Materiales, Facultad de Ciencias & Instituto Nicolás Cabrera Universidad Autonoma de Madrid, Madrid 28049, Spain.
[2] Universidad de Granada, Nanoparticles Trapping Laboratory, Research Unit Modeling Nature (MNat) and Department of Applied Physics, 18071 Granada, Spain.
[3] Universidad de Málaga, Department of Applied Physics II, 29071 Málaga, Spain.
[4] Biomedical Magnetic Resonance Laboratory-BMRL, Andalusian Public Foundation Progress and Health-FPS, 41092 Sevilla, Spain.
[5] Biomedical Research Institute of Málaga and Nanomedicine Platform (IBIMA-BIONAND Platform), University of Málaga, C/Severo Ochoa 35, 29590 Málaga, Spain.
[6] Biomedical Research Networking Center in Bioengineering, Biomaterials & Nanomedicine (CIBER-BBN), 28029 Madrid, Spain.
[7] Department of Physical Chemistry, Universidad Complutense de Madrid, 28040 Madrid, Spain.
*Corresponding authors: rul@ugr.es, frgamez@ucm.es

I. ADDITIONAL FIGURES

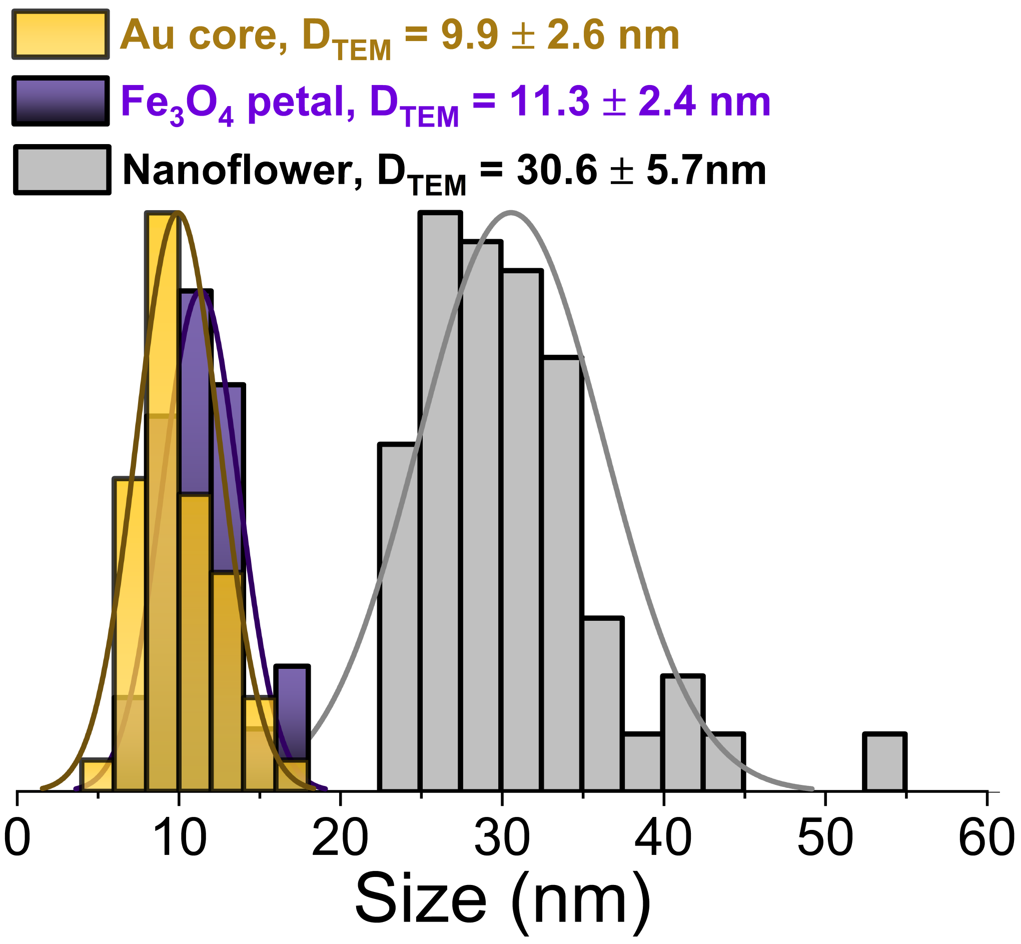

Fig. S 1. Size distribution of the NPs moieties (i. e., magnetite petals, gold core and whole NP), as obtained by the analysis of the TEM images.

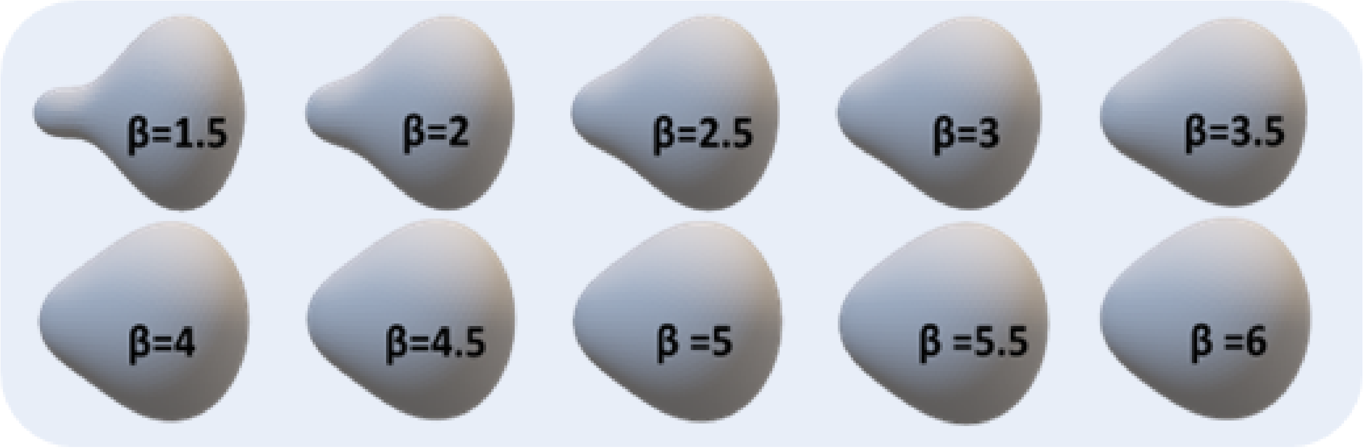

Fig. S 2. Theoretical models for the petals employed in the calculation of the scattering profiles of the nanoflowers.

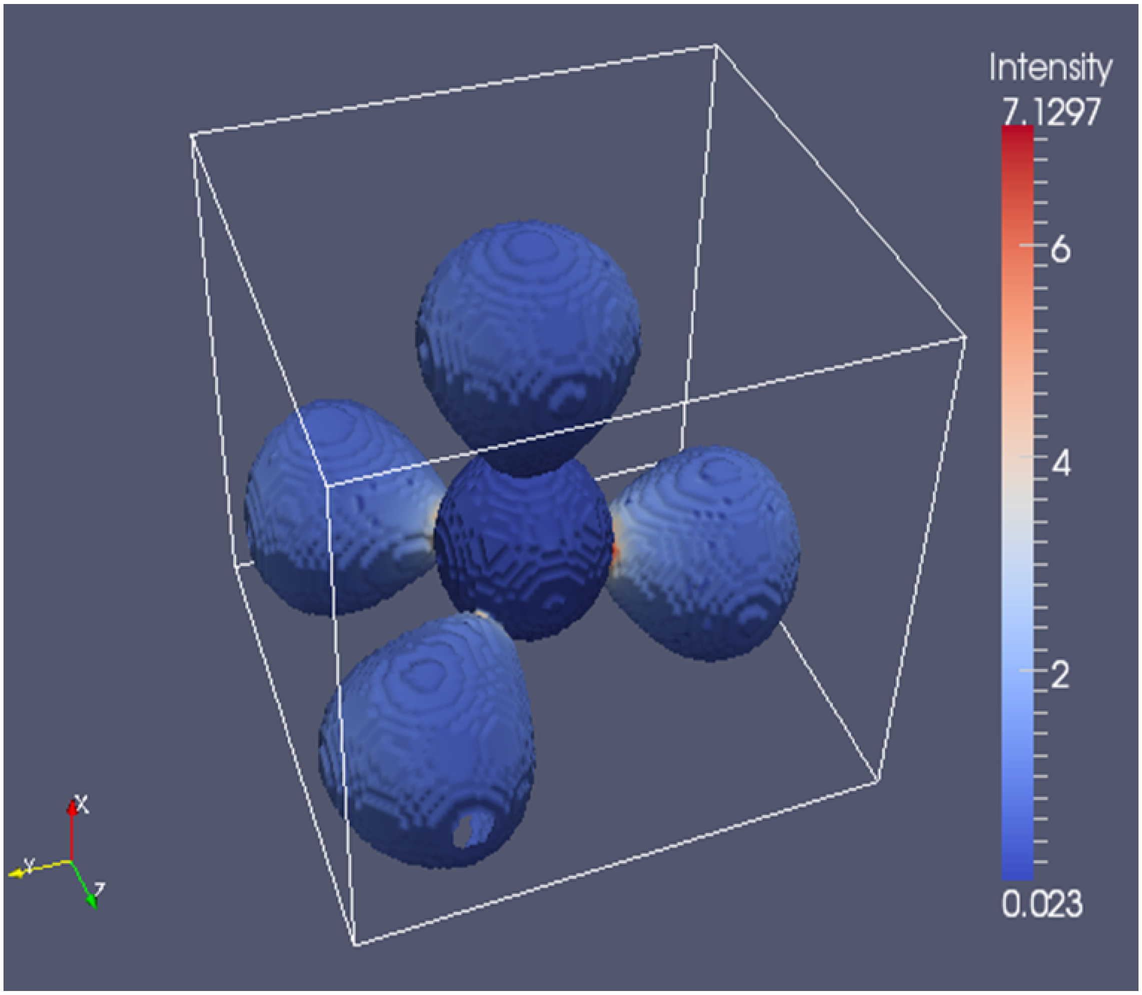

Fig. S 3. 3D plot of the calculated electric field intensity for the model T3 at 1064 nm.

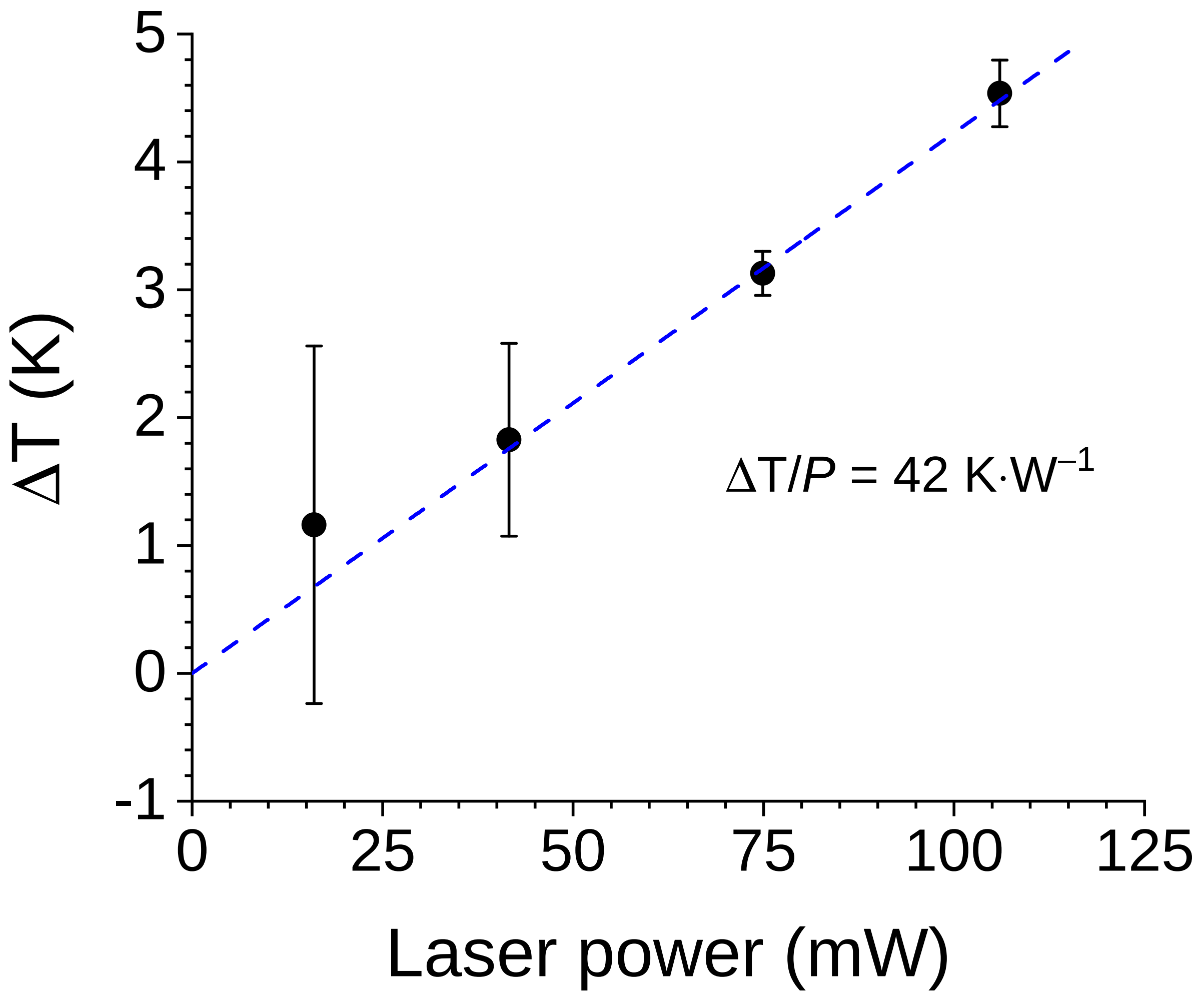

Fig. S 4. Calibration curve for evaluating the nanothermometers contribution to the observed temperature. See details in the Methods section.
